# Acute subdural hematoma in spontaneous intracranial hypotension: illustrative case and review of the literature

**DOI:** 10.1016/j.bas.2026.107439

**Published:** 2026-09-09

**Authors:** Manou Overstijns, Jakob Straehle, Katharina Wolf, Roberto Doria-Medina, Marco Bissolo, Amir El Rahal, Niklas Lützen, Horst Urbach, Jürgen Beck, Florian Volz

**Affiliations:** a Department of Neurosurgery, Medical Center University of Freiburg, Freiburg, Germany; b Department of Neuroradiology, Medical Center University of Freiburg, Freiburg, Germany

**Keywords:** Spinal CSF leak, Spontaneous intracranial hypotension, Acute subdural hematoma, Cerebrospinal fluid leak, Spinal longitudinal epidural collection (SLEC), Ventral dural tear

## Abstract

**Introduction:**

Spinal cerebrospinal fluid (CSF) leaks cause spontaneous intracranial hypotension (SIH) and are a recognized cause of chronic subdural hematoma (cSDH). In contrast, acute subdural hematoma (aSDH) without trauma as the initial manifestation of a spinal CSF leak appears to be exceedingly rare.

**Research question:**

Can a primary non-traumatic aSDH represent the first manifestation of a spinal CSF leak.

**Material and methods:**

We report an illustrative case of a 52-year-old man presenting with a non-traumatic aSDH requiring emergency surgical evacuation together with a PRISMA-based literature review of the literature on subdural hematomas associated with SIH/spinal CSF leaks. PubMed and Embase databases were systematically screened. Diagnostic workup included cranial imaging, digital subtraction angiography, spinal MRI, and dynamic subtraction myelography.

**Results:**

The literature review identified 14 eligible publications. SIH-associated subdural hematomas were predominantly chronic or mixed acute-on-chronic in nature, whereas primary acute presentations were exceedingly uncommon. No documented case of a primary non-traumatic aSDH caused by a spinal CSF leak was identified. In the presented case, repeated cranial vascular imaging revealed no vascular pathology. The patient developed recurrent subdural hematomas requiring multiple surgical interventions. Subsequent spinal imaging demonstrated a ventral CSF leak at T5/6 caused by a discogenic microspur. Minimally invasive transdural surgical repair resulted in complete clinical and radiological resolution.

**Discussion and conclusion:**

Primary non-traumatic aSDH may represent the first manifestation of a spinal CSF leak. In unexplained or recurrent subdural hematomas, dedicated spinal CSF leak diagnostics should be considered, as definitive closure of the underlying leak represents the causal treatment.

## Highlights

• Primary aSDH can be first manifestation of spinal CSF leak.
• Non-traumatic aSDH requires exclusion of SIH and spinal CSF leaks.
• Recurrent SDH without cause should prompt spinal imaging workup.
• Dynamic myelography enables precise localization of ventral leaks.
• Surgical CSF leak closure leads to complete and durable resolution.

## Non-standard abbreviations and acronyms:

• **SIH**: Spontaneous intracranial hypotension
• **CSF**: Cerebrospinal fluid
• **aSDH**: Acute subdural hematoma
• **cSDH**: Chronic subdural hematoma
• **SLEC**: Spinal longitudinal epidural collection
• **DSM**: dynamic digital subtraction myelography

## 1 Introduction

Spontaneous intracranial hypotension (SIH) has an estimated annual incidence rate of around 4 per 100,000 and is caused by spontaneous cerebrospinal fluid (CSF) loss from a spinal CSF leak (Schievink et al., 2022). Spinal CSF leaks are categorized into three main subtypes: ventral dural tears (Type 1), lateral dural tears near the exiting nerve root sleeve (Type 2) and CSF–venous fistulas causing uncontrolled CSF outflow into the epidural venous system (Type 3) (Schievink et al., 2016). Chronic subdural hematoma (cSDH) occurs in 25.9% to 43.5% (Beck et al., 2014; El Rahal et al., 2023; Overstijns et al., 2025) of patients with SIH. Although the association between SIH and cSDH is well recognized, the development of an acute subdural hematoma (aSDH) as a primary manifestation of SIH remains exceedingly rare (Beck et al., 2014; Overstijns et al., 2025; Tanaka et al., 2016). Although the association between SIH and cSDH is well recognized, SIH-associated subdural hematomas reported in the literature are predominantly chronic, bilateral, or mixed-density lesions. Acute SDH has only rarely been described and has generally occurred in the setting of a pre-existing chronic subdural collection or following cranial intervention (Kuramae et al., 2011; Perez-Vega et al., 2020). An aSDH after an iatrogenic spinal leak has bean described, however a primary spontaneous, non-traumatic non-iatrogenic aSDH as the initial manifestation of a previously unrecognized spontaneous spinal CSF leak appears to be exceptionally rare (Sciubba et al., 2005).

We therefore present an illustrative case of a non-traumatic aSDH as the first manifestation of a spinal CSF leak requiring repeated neurosurgical intervention before definitive diagnosis and treatment of the underlying leak. In addition, we performed a systematic review of the literature on subdural hematomas associated with SIH/spinal CSF leaks to contextualize this rare acute presentation within the broader spectrum of SIH-related subdural bleeding.

## 2 Methods

### 2.1 Case report

Clinical, radiological, surgical, and follow-up data of the presented patient were retrospectively reviewed. The patient provided informed consent for publication. Institutional ethical approval was obtained (Ethical approval code: 24-1357_1-S1).

### 2.2 Review of the literature

The comprehensive review of the literature was conducted in accordance with the Preferred Reporting Items for Systematic Reviews and Meta-Analyses (PRISMA) statement. PubMed and Embase databases were systematically searched for studies form 01.01.2000 until 31.03.2026 reporting subdural hematomas associated with spontaneous intracranial hypotension or spinal cerebrospinal fluid leaks using a standardized search term (Supplemental material 1). Cohort Studies, cases series, and case reports involving adult patients with SIH/spinal CSF leaks and associated subdural hematomas were included. Traumatic subdural hematomas and postoperative CSF leaks were excluded. Titles, abstracts, and full texts were screened for eligibility, and any uncertainties regarding study inclusion were resolved by discussion between two authors (MO, FV). Data regarding clinical presentation, hematoma characteristics, treatment strategies, and outcomes were extracted for qualitative analysis.

## 3 Results

### 3.1 Review of the literature

The literature search identified 317 records through PubMed and Embase database screening (Fig. 1). After title and abstract review, 286 studies were excluded because they did not report subdural hematomas associated with spontaneous intracranial hypotension or spinal CSF leaks. The remaining 31 articles underwent full-text assessment for eligibility. Following exclusion of 17 publications after full-text review, 14 studies were included in the final qualitative synthesis (Supplemental material 2).

**Fig. 1:**
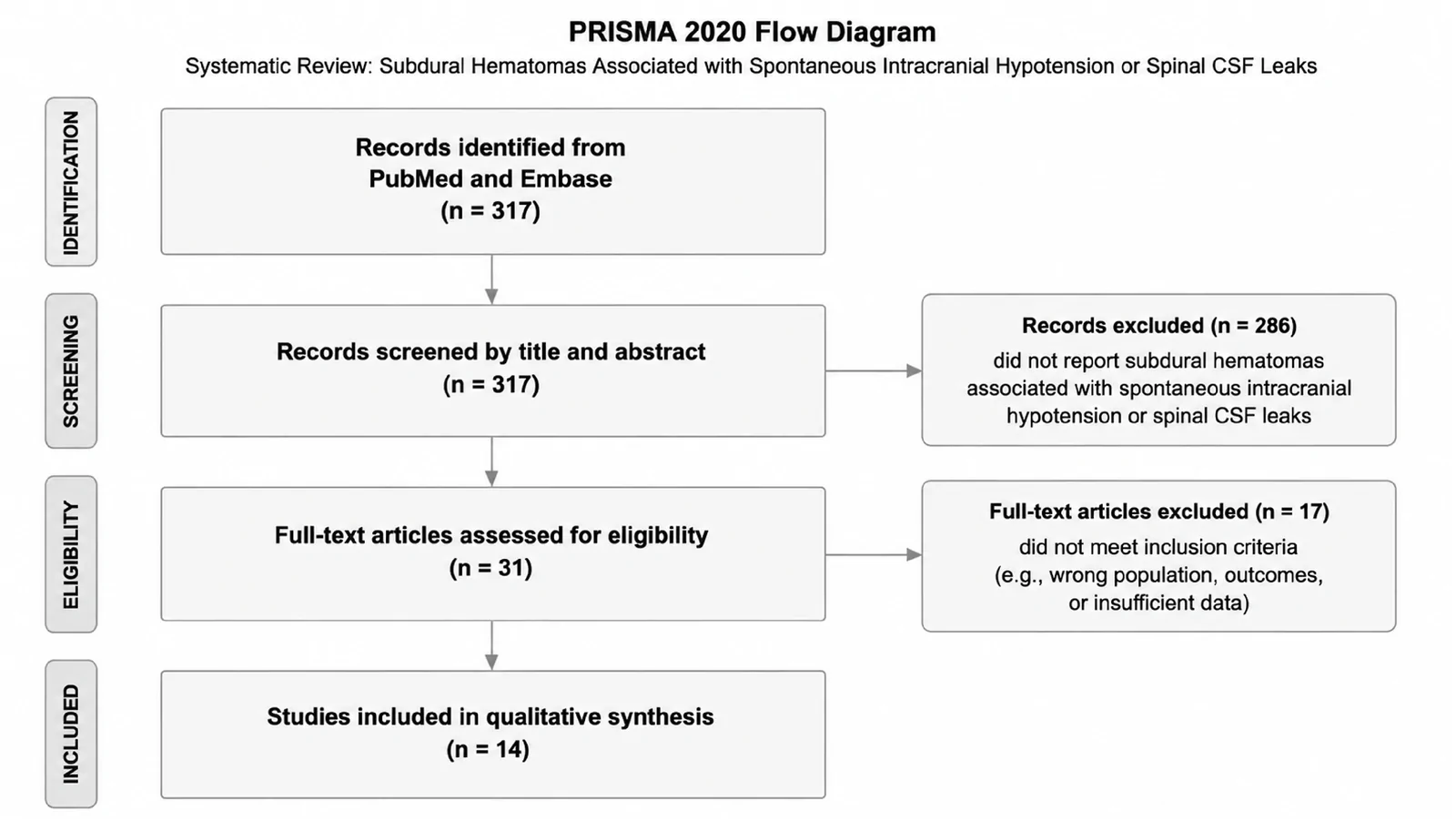
Flowchart of the literature search.

## 4 Illustrative case

A 52-year-old man without pre-exisitng medical condition presented to the emergency department with sudden onset of severe, but not orthostatic, headache and nonspecific visual disturbances. There were no history or external signs of trauma. Cranial CT demonstrated a right hemispheric aSDH (coronal width of 12 mm), with severe mass effect and midline shift, prompting emergency neurosurgical admission for immediate evacuation (Fig. 2A). The patient denied antiplatelet or anticoagulant medication intake, and laboratory testing showed no evidence of coagulopathy. CT angiography demonstrated no vascular abnormality. Because of the unusual spontaneous aSDH, subsequent catheter digital subtraction angiography was performed and likewise demonstrated no aneurysm, arteriovenous malformation, or dural arteriovenous fistula. There was no imaging evidence of cerebral venous sinus thrombosis or relevant venous outflow obstruction on the available vascular imaging. There was no clinical history or cranial imaging finding suggestive of an underlying malignancy. The patient denied the intake of antiplatelet or anticoagulant medication and laboratory analysis revealed no evidence of coagulopathy. During the initial examination the patient was completely awake and conscious with a Glasgow Coma Scale score of 15. However, he deteriorated rapidly while being transferred to the operating room and presented with a newly dilated right sided pupil just before skin incision. Immediate emergency craniotomy with hematoma evacuation was performed. Postoperatively, the pupils normalized and the patient was extubated immediately after surgery without neurological deficits. The postoperative CT showed adequate hematoma evacuation (Fig. 2B).

**Fig. 2:**
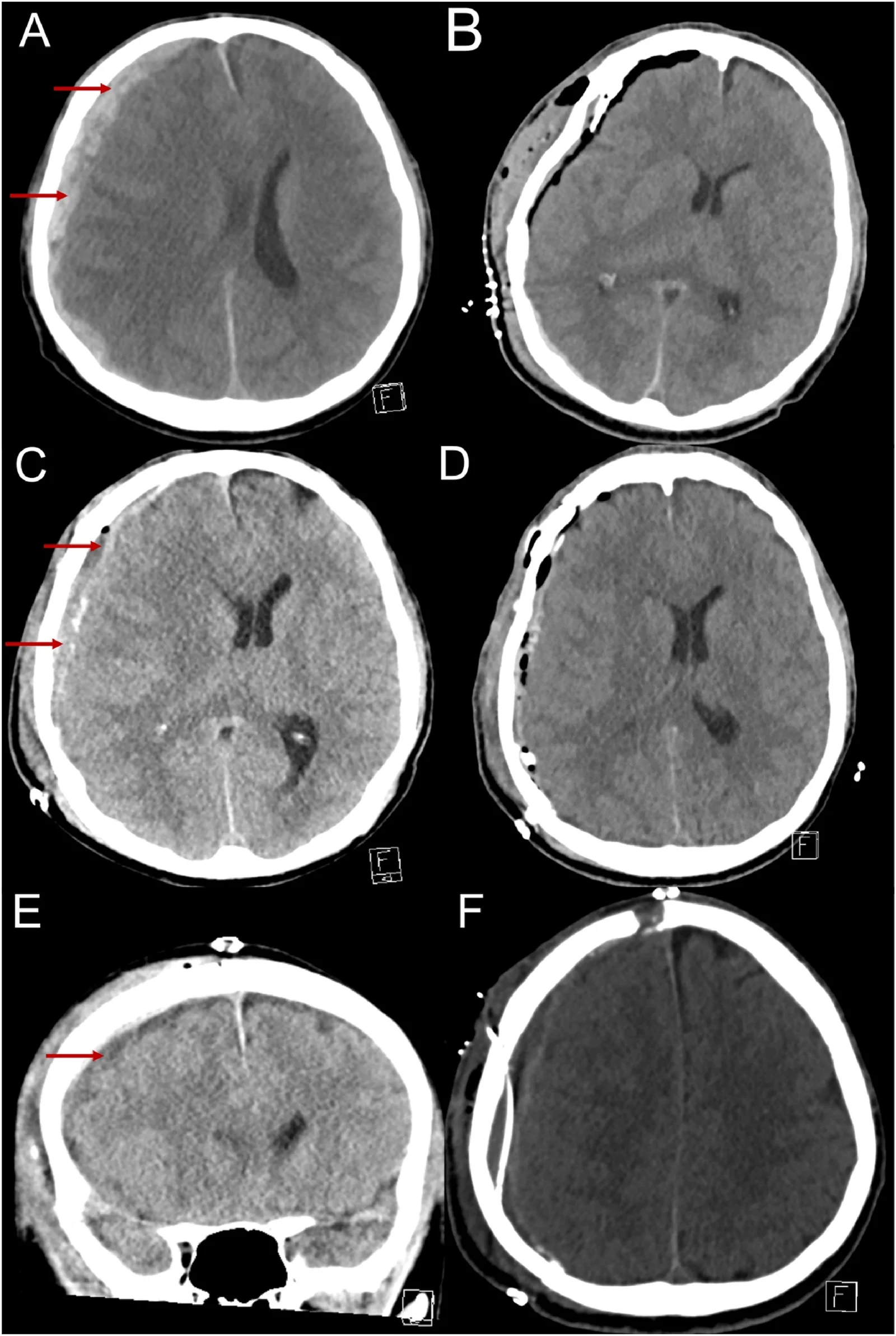
A): Initial axial CT showing an acute subdural hematoma on the right hemisphere with mass-effect and severe midline-shift. B) Postoperative CT showing sufficient hematoma evacuation and receding mass effect. C) Second preoperative axial CT showing a recurrent right-sided cSDH containing acute hemorrhagic components, again with clear midline-shift. D) Axial CT after the second craniotomy showing a sufficient hematoma evacuation E) Coronal CT showing again a recurrent hematoma on the right hemisphere, this time with a more chronic appearance. F) CT after the twist-drill craniostomy showing the correct position of the subdural drainage.

Ten days later, he again developed a severe headache and left-sided hemiparesis. CT imaging showed a cSDH with acute components, again with significant midline shift (Fig. 2 C). The patient underwent a second emergency craniotomy with hematoma evacuation (Fig. 2 D). Postoperatively, the hemiparesis completely resolved and he was discharged 6 days later, again without a neurological deficit. Three days later, the patient presented for the third time with a severe headache. CT imaging again showed a rightsided cSDH with moderate midline shift (Fig. 2 E). The patient underwent twist-drill craniostomy at the frontal eminence with placement of a subdural drain the same day. Postoperative CT confirmed a correct subdural position of the drain. The drain was removed the next day, and the patient remained without neurological deficits (Fig. 2 F).

Given the initial presentation of an aSDH without trauma, repeated exclusion of a vascular pathology combined with the unusual hematoma recurrence pattern, a focused spinal and cranial MRI imaging was performed. This revealed bilateral pachymeningeal enhancement, brain sagging, and small residual right sided cSDH (Fig. 3A and B) resulting in a Bern SIH score of 6 points (representing a high probability for a spinal CSF leak) (Dobrocky et al., 2019). Spinal MRT showed a “spinal longitudinal extradural CSF collection” (“SLEC positive”) (Fig. 3C and D), indicating a spinal dural tear. For exact localization of the spinal CSF leak, a dynamic digital subtraction myelography (DSM) in prone position was performed, demonstrating a ventral (Type 1) leak at T5/6 (Fig. 3 E), associated with a discogenic microspur (Fig. 3 F).

**Fig. 3:**
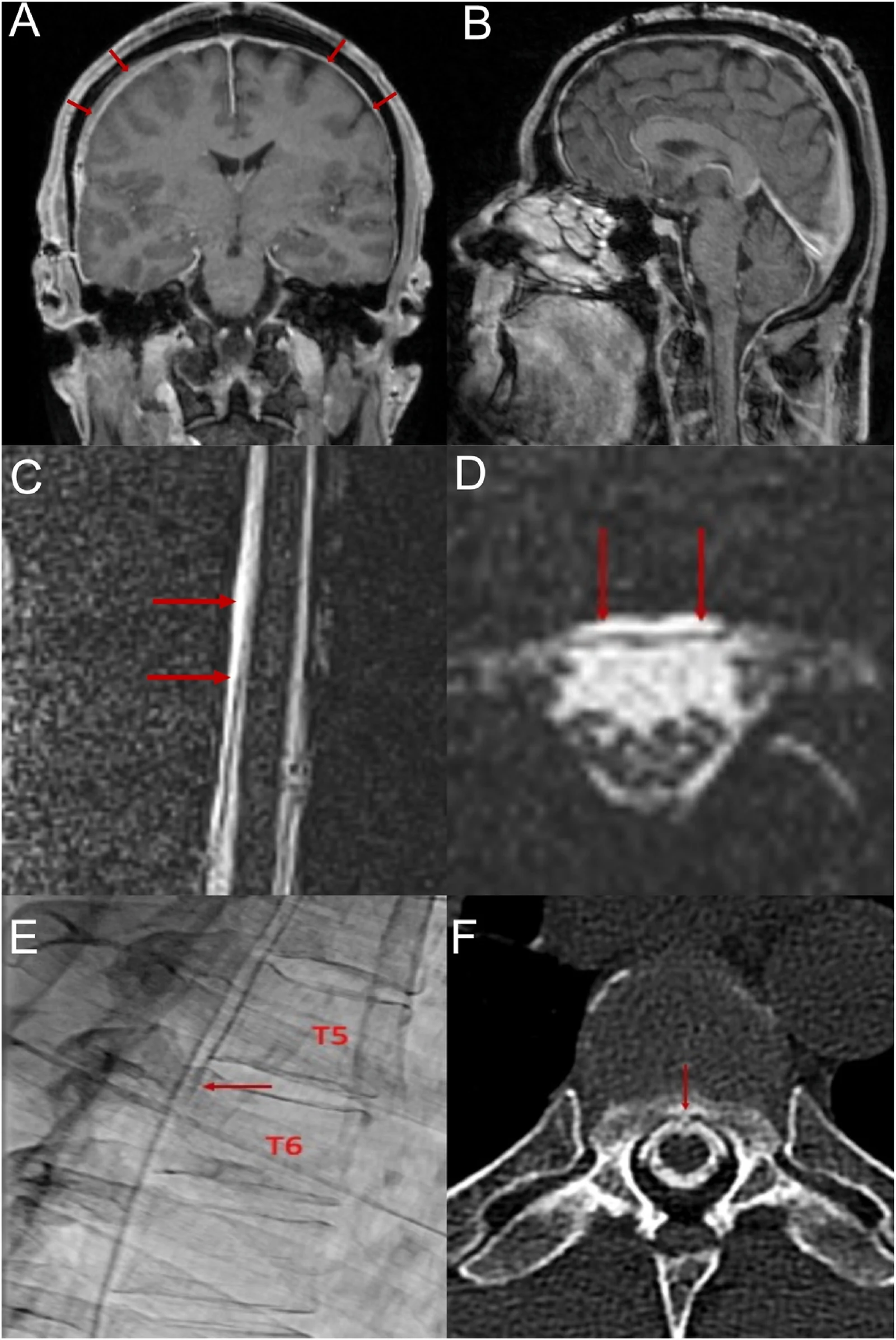
A-B): MRI of the head with contrast showing brain sagging, bilateral meningeal enhancement (red arrows) and a small residual right sided cSDH resulting in a SIH-score of 6. C-D) Fat-saturated and heavily T2-weighted MRI of the spine showing a ventral Spinal Longitudinal extradural CSF-Collection (SLEC). E) Dynamic Digital Subtraction Myelography (DSM) showing a ventral leak at of T5/6. F) Axial CT-Myelography following DSM shows a bony microspur.

The patient underwent minimally invasive microsurgical, transdural disconnection of fistula membranes and transdural funnels, removal of the osseous microspur and sandwich-technique closure of the ventral dural defect (Fig. 4A–D) (Häni et al., 2024) (Beck et al., 2023), (Volz et al., 2024a).

**Fig. 4:**
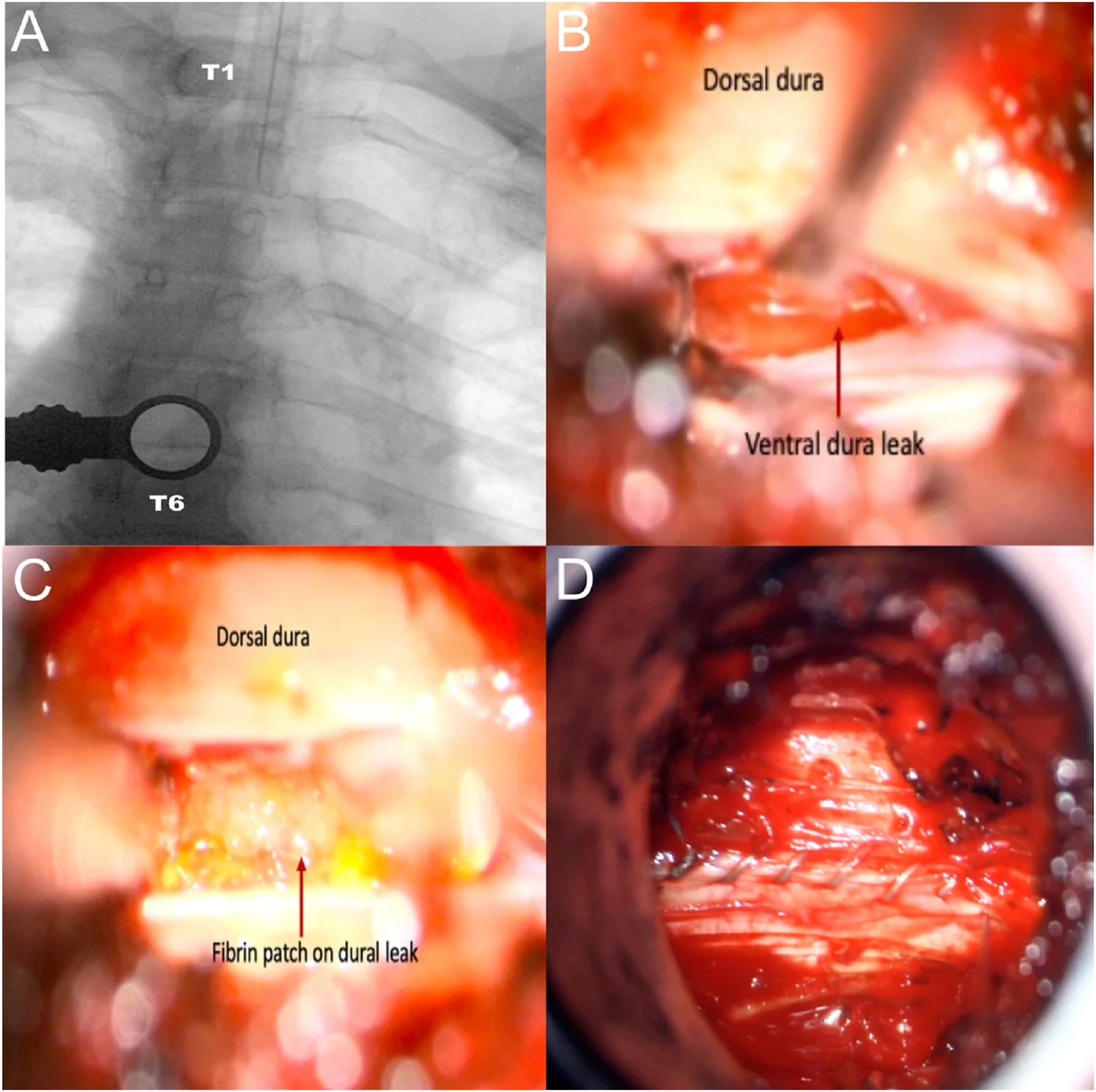
A) intraoperative fluoroscopy confirming the correct working trajectory through the non-expandable tubular retractor centered just above the disc space T5-6. Note the deliberately oblique view directly through the retractor. B) Intraoperatve disconnection of fistula membranes (“funnels”) of the ventral leak at T5/6. C) Sandwich technique closure of the defect. D) Dorsal durotomy closure using running sutures.

Postoperatively, the patient experienced mild transient rebound hypertension headaches and a transient right-leg dysesthesia without motor deficit for two days, both of which resolved spontanously. At the six week follow-up, the patient was asymptomatic. MRI of the brain and spine showed clearly reduced brain sagging, and complete regression of the subdural hematoma and extrathecal CSF (now “SLEC negative”) (Fig. 5A–C). Postoperative CT showed the small bone defect of the hemilamina T5 right (Fig. 5D) after unilateral keyhole fenestration (Volz et al., 2024a).

**Fig. 5:**
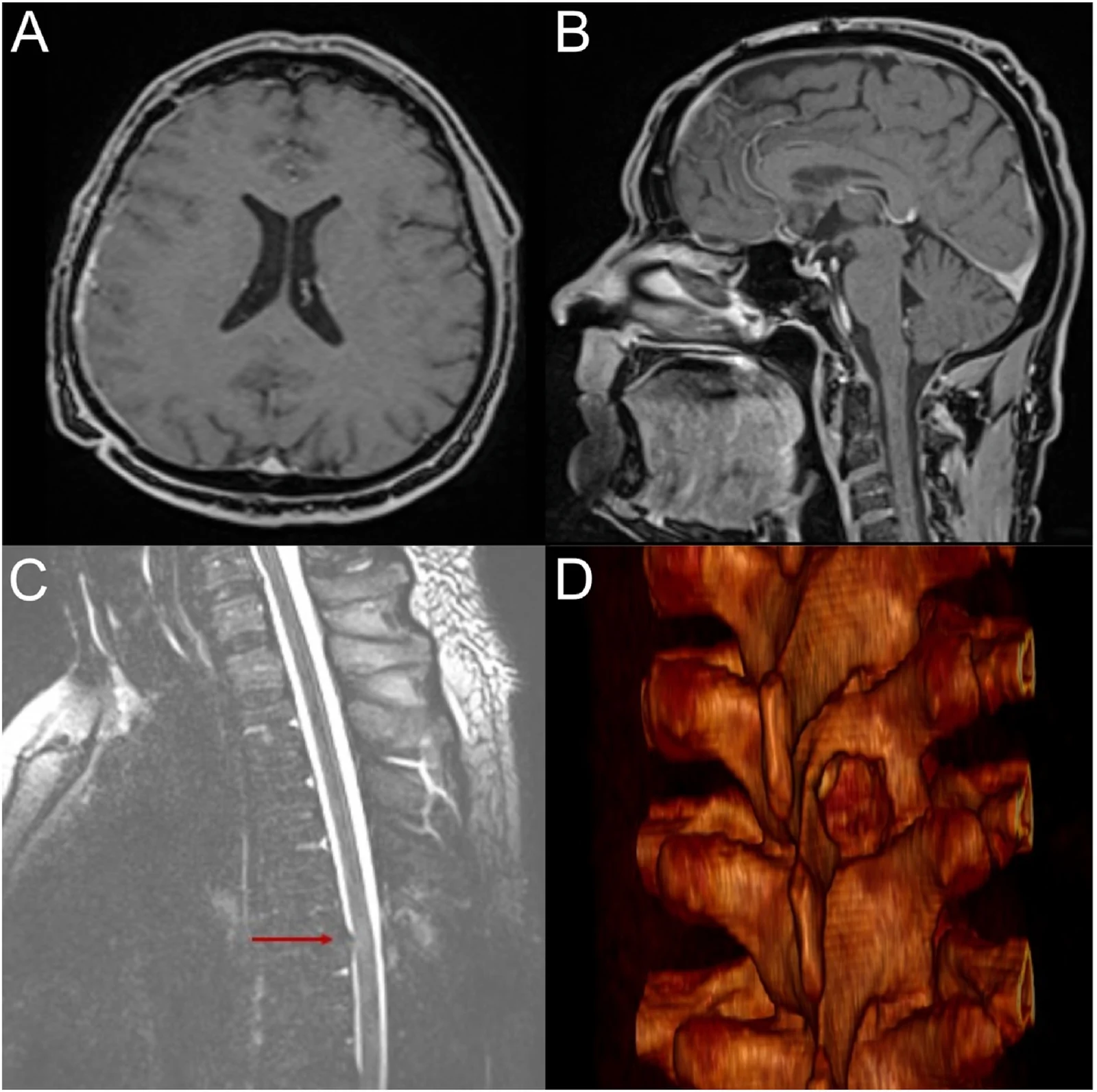
A-B) 6 weeks postoperative MRI showing complete regression of subdural hematoma and obvious improvement of the brain sagging. C) Postoperative MRI of operated level T5/6 shows normal findings without relevant spinal cord compression or myelopathy, and disappearance of the Spinal Longitudinal Epidural Collection (SLEC). D) Postoperative CT showing the limited unilateral bone defect of right T5 hemilamina after the keyhole fenestration.

## 5 Discussion

The novel observation of the present case is that a primary spontaneous, non-traumatic and non-iatrogenic aSDH may represent the first manifestation of a previously unrecognized spontaneous spinal CSF leak. Although the association between SIH and subdural hematoma is well established, the available literature predominantly describes chronic, bilateral, or mixed acute-on-chronic subdural hematomas (Beck et al., 2014; El Rahal et al., 2023). Acute subdural hematoma has also been reported following iatrogenic spinal CSF leakage after dural injury during spine surgery (Sciubba et al., 2005; Lu et al., 2002). In our systematic review, we identified no previous report of a primary spontaneous, non-traumatic or non-iatrogenic aSDH occurring as the initial manifestation of a spontaneous spinal CSF leak without prior cranial or spinal intervention.

The literature review revealed that subdural hematomas associated with spontaneous spinal CSF leaks were predominantly chronic or mixed acute-on-chronic in nature. No case with an spontaneous, non-traumatic acute subdural hematoma as initial symptom was reported. Several cohort studies demonstrated that approximately 17–43% of patients with SIH develop concomitant subdural hematomas, most frequently bilateral chronic subdural hematomas (Overstijns et al., 2025; Wang et al., 2026; Kim et al., 2021). In a recent cohort study, among patients with SIH-related cSDH, 58.8% had type 1 ventral leaks, 15.3% had type 2 lateral leaks, and 25.9% had type 3 CSF-venous fistulas; conversely, cSDH prevalence was 31% in type 1, 22% in type 2, and 43% in type 3 leaks (Overstijns et al., 2025). Orthostatic headache remained the most common presenting symptom, reported in approximately 76–98% of patients, although atypical presentations without clear positional headache were repeatedly described (El Rahal et al., 2023), (Dehnavi et al., 2026) (D'Antona et al., 2021), (Luetzen et al., 2021).

Across the reviewed literature, recurrent, rapidly progressive, or otherwise unexplained subdural hematomas, particularly in younger patients without trauma or coagulopathy, were repeatedly emphasized as important clues to an underlying spinal CSF leak. (El Rahal et al., 2023; Overstijns et al., 2025).

Although the link between spinal CSF leaks and cSDH is well established (Beck et al., 2014; Overstijns et al., 2025; Takahashi et al., 2016), the presentation of an aSDH prior to any intervention remains very rare (Beck et al., 2014; Overstijns et al., 2025; de Noronha et al., 2003). The acute SDHs identified in our review occurred in the context of a pre-existing subdural collection, following placement or removal of a subdural drain for a pre-existing cSDH, or after a iatrogenic dural CSF leak following spinal surgery (Perez-Vega et al., 2020; Sciubba et al., 2005; Chotai et al., 2013; Wen et al., 2022).

In spinal CSF leak related cSDH, recurrence appears to depend most on whether the underlying spinal leak is definitively closed. In a surgical cohort, once the leak was surgically sealed, no cSDH recurred during follow-up, regardless of whether the hematoma had been managed conservatively or with cranial evacuation first (Overstijns et al., 2025). Surgical treatment of the spinal CSF leaks via minimally invasive approaches is a safe and effective technique that yields, in experienced centers, excellent outcomes with closure rates of around 95% and very low morbidity (Beck et al., 2023; Petutschnigg et al., 2025; El Rahal et al., 2025). By contrast, epidural blood patching can improve symptoms and sometimes control the leak, but recurrence or persistence can be as high as 48% (Hazama et al., 2023; Hashizume et al., 2013). Middle meningeal artery embolization may help reduce recurrence of non-acute SDH in general, but it does not treat the causal spinal CSF leak, although specific data on patients with spinal CSF leakage is lacking. The broader SDH literature and consensus documents describe MMA embolization as a recurrence-reducing adjunct, not a substitute for fixing the underlying SIH lesion (Siddiq et al., 2025; Maciaszek et al., 2025). In SIH-associated cSDH, this means MMA embolization may support hematoma control, but durable prevention of recurrence still depends on definitive leak closure.

The presumed pathophysiological mechanism underlying cSDH formation in patients with spinal CSF leaks is that continuous spinal CSF loss reduces intracranial CSF volume, resulting in brain sagging and traction on bridging veins, ultimately leading to subdural fluid collections and hemorrhage (Beck et al., 2014; El Rahal et al., 2023). This proposed mechanism is supported by radiological markers such as diffuse pachymeningeal enhancement, venous engorgement, all of which were present in this patient (Dobrocky et al., 2019; Luetzen et al., 2021; Miller and Nader, 2014; Yamashima and Friede, 1984). However, rupture of cortical and meningeal arteries or arterialized veins can also occur following loss of CSF volume, therefore an aSDH should not automatically argue against SIH as the underlying etiology (Miller and Nader, 2014; Rengachary and Szymanski, 1981). In patients presenting with aSDH without a history of trauma, alternative etiologies must be systematically excluded including vascular etiology (Akioka et al., 2007). These include ruptured intracranial aneurysms, arteriovenous malformations, dural arteriovenous fistulas, cerebral venous pathology, coagulopathy, antithrombotic therapy, and less commonly intracranial malignancy. Although most aSDH are traumatic, non-traumatic cases have been associated with ruptured intracranial aneurysms, arteriovenous malformations, dural arteriovenous fistulas, and venous hypertension (Orde, 2019; Beucler et al., 2019; Coombs et al., 2014). This case argues that spinal CSF leaks should be added to this differential diagnosis list. Especially in unusual cases or when a vascular cause has been clearly ruled out dedicated spinal CSF-leak diagnostics should be performed, including MRI of head and complete spine and potentially dynamic myelography techniques (Volz et al., 2024b).

## 6 Limitations

This study has several limitations. First, the available literature on SIH-associated subdural hematomas consists predominantly of retrospective series and isolated case reports, resulting in substantial heterogeneity regarding diagnostic workup, treatment strategies, and follow-up duration. Second, publication bias toward unusual or severe presentations cannot be excluded. Finally, due to the rarity of acute hemorrhagic presentations, quantitative subgroup analysis of primary aSDH was not feasible.

## 7 Conclusion

This illustrative case and literature review demonstrate that spinal CSF leaks should be considered among the differential diagnoses in patients presenting with non-traumatic or recurrent subdural hematomas, even in the absence of classical orthostatic headache. Although SIH-associated subdural hematomas are predominantly chronic in nature, rare spontaneous and non-traumatic acute presentations may occur that require emergency neurosurgical intervention. Recurrent or atypical hematoma patterns should therefore prompt dedicated spinal CSF leak diagnostics, including cranial and spinal MRI as well as dynamic myelographic techniques when indicated. While surgical evacuation of the hematoma may be necessary in symptomatic patients, definitive and durable treatment ultimately depends on successful closure of the underlying spinal CSF leak, resulting in resolution of both intracranial and spinal manifestations.

## Consent to participate

Written informed consent of patient acquired.

## Availability of data and material

On request.

## Code availability

Not applicable.

## Consent for publication

Consent of all authors.

## Author's contributions

Manou Overstijns, Jakob Straehle, Jürgen Beck, and Florian Volz conceived and designed the study. Data acquisition was performed by Manou Overstijns, Katharina Wolf, Roberto Doria-Medina, Amir El Rahal, and Niklas Lützen. Infrastructure and technical support were provided by Horst Urbach, Niklas Lützen, Jürgen Beck, and Florian Volz. Data analysis and interpretation were carried out by Manou Overstijns, Katharina Wolf, Jakob Straehle, Niklas Lützen, Florian Volz, and Jürgen Beck. Graphical illustrations were prepared by Roberto Doria-Medina. The manuscript was drafted by Manou Overstijns and Florian Volz with contributions from all authors. All authors critically revised the manuscript and approved the final version.

## Ethics declaration

Written informed consent to take part in the study and to publish the article has been obtained from all participants or their legal representatives. The privacy rights of participants have been observed.

This study was performed in compliance with relevant laws, regulatory frameworks and guidelines where the research took place. This study was approved by the University Hospital Freiburg. (Approval No. 24-1357_1-S1)

This research follows the CARE guidelines and the CARE checklist.

## Submission declaration and verification

The authors confirm that the work described in this manuscript has not been published previously, except where permitted in the form of an abstract, published lecture, or academic thesis, and that it is not currently under consideration for publication elsewhere. All authors have reviewed and approved the manuscript and consent to its submission and publication. Where applicable, the responsible institutional authorities have also approved the work.

The authors further confirm that, if accepted, the manuscript will not be published elsewhere in the same form, in English or in any other language, including electronically, without the written consent of the copyright holder.

The authors acknowledge that the manuscript may be screened using Crossref Similarity Check or other originality and duplicate-publication detection software to verify compliance with the journal's publication policies.

## Funding or financial support

None.

## Declaration of competing interests

The authors declare that they have no known competing financial interests or personal relationships that could have appeared to influence the work reported in this paper.
