# Supplementary material for: Acute subdural hematoma in spontaneous intracranial hypotension: illustrative case and review of the literature
Source: Brain Spine. 2026 Sep 9;6:107439. doi: 10.1016/j.bas.2026.107439 (PMC13634392; doi:10.1016/j.bas.2026.107439)
Supplement: Supplementary file 1 [file mmc1.docx]

Supplemental material 1:

Search strategy;

("Intracranial Hypotension"[Mesh] OR "Cerebrospinal Fluid Leak"[Mesh] OR "Spinal Cerebrospinal Fluid Leak" OR "spontaneous intracranial hypotension" OR "spinal CSF leak") AND ("Hematoma, Subdural"[Mesh] OR "subdural hematoma" OR "acute subdural hematoma" OR "chronic subdural hematoma")

Supplemental material 2:

Studies included in the review

| **Study** | **Design / population** | **Hematoma type / laterality** | **Symptoms / orthostatic headache** | **Vascular studies** | **Brain MRI (SIH)** | **Spinal imaging / leak level** | **SDH treatment** | **Leak treatment** | **Recurrence / outcome** |
| --- | --- | --- | --- | --- | --- | --- | --- | --- | --- |
| de Noronha et al., 2003 | Case series; SIH+SDH | Large SDH; detailed type/laterality NR | Postural headache; acute deterioration in some | NR | SIH MRI abnormalities; individual findings NR | Leak localization NR | Urgent drainage in selected cases | NR | Individual long-term outcome NR |
| Beck et al., 2014 | Cohort; non-geriatric cSDH | cSDH; unilateral/bilateral | NR | NR | NR | Spinal MRI + myelography; levels NR | cSDH surgery | NR in aggregate report | NR |
| Takahashi et al., 2016 | Retrospective series; SIH+cSDH | cSDH; laterality NR | Orthostatic headache important diagnostic feature | NR | Diffuse meningeal enhancement | NR | Conservative or hematoma irrigation | EBP in selected patients | Further treatment in some; good outcomes |
| Tanaka et al., 2016 | Retrospective SIH cohort | SDH/subdural collections; type/laterality NR | Headache common; atypical patterns described | NR | Pachymeningeal enhancement frequent | NR | NR | NR | NR |
| Kim et al., 2021 | Retrospective; non-traumatic SDH | Unilateral and bilateral SDH | Orthostatic headache in subset | NR | NR | CT myelography; leak in many; levels individualized | Surgery in selected cases | Targeted EBP | Some recurrences; additional treatment as needed |
| D'Antona et al., 2021 | Systematic review/meta-analysis; SIH | SDH-specific type/laterality NR | Orthostatic headache predominant overall | NR | Pachymeningeal enhancement and other SIH signs | Various modalities; SDH-specific level NR | NR for SDH subgroup | EBP/other SIH treatments | SDH-specific outcome NR |
| Kuramae et al., 2011 | Case report; SIH+SDH | Bilateral cSDH; postoperative aSDH | Headache, non-orthostatic; deterioration | NR | Diffuse meningeal enhancement | CT myelography; cervical leak | Burr holes, then emergency craniotomy | EBP, then surgical closure | Resolution; neurologically intact |
| Chotai et al., 2013 | Case report; SIH+SDH | Bilateral cSDH; post-drain hemorrhagic deterioration | Headache; orthostatic component unclear | Angiography/CTA negative | Brain sagging/downward displacement | CT myelography; cervical leak | Bilateral burr holes; emergency cranial surgery | Positioning + EBP | Substantial recovery |
| Perez-Vega et al., 2020 | Case report; SIH+SDH | Bilateral subacute/chronic SDH; acute recurrence | Orthostatic headache; later deterioration | NR | Pachymeningeal enhancement, venous engorgement, pituitary enlargement, brain sagging | CT myelography; leak not localized | Repeated evacuation/drainage | EBP | Recurrence during drain removal; favorable follow-up |
| Overstijns et al., 2025 | Cohort; SIH+cSDH | cSDH; predominantly bilateral | Focal deficits/seizures/reduced consciousness in subset; OH NR for subgroup | NR | Bern SIH-score features | Brain/spinal MRI + dynamic myelography/CTM; type 1-3 leaks | Conservative or evacuation | Definitive targeted leak closure | No cSDH recurrence after leak closure |
| Wang et al., 2026 | Cohort; SIH; SDH subgroup | Predominantly bilateral SDH | SDH-specific symptoms NR | NR | Dural enhancement associated with SDH | Specific leak localization NR | NR | NR | Treatment/outcome not primary focus |
| Dehnavi et al., 2026 | Systematic review; bilateral SIH-SDH | Bilateral; chronic/mixed-density | Variable; OH often reported | Variable/NR | SIH findings across studies | Variable; leak level often incomplete | Conservative or evacuation | EBP/other leak-directed therapy | Heterogeneous; some patient-level outcomes |
| Kim et al., 2019 | Case-control; bilateral collections in SIH | Bilateral hygroma/cSDH | Patient-level headache NR | NR | Brain sagging and pseudo-SAH associated with SIH | NR | Burr hole in selected patients | EBP | Generally favorable outcomes |

Abbreviations: NR, not reported; OH, orthostatic headache; aSDH, acute subdural hematoma; cSDH, chronic subdural hematoma; CSF, cerebrospinal fluid; CTM, CT myelography; EBP, epidural blood patch; SDH, subdural hematoma; SIH, spontaneous intracranial hypotension.
