# Supplementary material for: Acute subdural hematoma in spontaneous intracranial hypotension: illustrative case and review of the literature
Source: Brain Spine. 2026 Sep 9;6:107439. doi: 10.1016/j.bas.2026.107439 (PMC13634392; doi:10.1016/j.bas.2026.107439)
Supplement: Supplementary file 2 [file mmc2.docx]

**Structured Literature Review**

**Title**
Acute Subdural Hematoma in Spontaneous Intracranial Hypotension: Review of the Literature

**Objective**
To review the available literature on subdural hematomas associated with spontaneous intracranial hypotension (SIH) or spinal cerebrospinal fluid (CSF) leaks, with particular focus on acute and non-traumatic presentations.

**Main Findings**
The literature confirms that the association between SIH and chronic subdural hematoma (cSDH) is well established, with reported incidences ranging from 17% to 43% in larger SIH cohorts. In contrast, acute subdural hematoma (aSDH) in the setting of SIH appears to be exceedingly rare. Reported acute hemorrhagic deteriorations predominantly occurred following surgical drainage procedures or rapid intracranial pressure changes in patients with pre-existing chronic subdural collections.

Importantly, no previous report describing a spontaneous, non-traumatic aSDH as the initial manifestation of a spinal CSF leak was identified in the reviewed literature.

**Synthesis of the Literature**
The available evidence suggests that continuous spinal CSF loss leads to intracranial CSF volume depletion, resulting in brain sagging and traction on bridging veins, thereby predisposing patients to subdural fluid collections and hemorrhage. While orthostatic headache remains the classical clinical presentation of SIH, atypical presentations without positional symptoms have repeatedly been described.

In patients presenting with unexplained or recurrent non-traumatic subdural hematomas, spinal CSF leak diagnostics should be considered after exclusion of intracranial vascular pathology. MRI of the brain and spine as well as dynamic myelographic techniques are essential for accurate diagnosis and localization of the underlying leak.

Therapeutically, evacuation of the subdural hematoma alone may provide temporary symptom relief but does not address the underlying pathology. Durable resolution depends on definitive closure of the spinal CSF leak.

**Conclusion**
This literature review demonstrates that spontaneous non-traumatic aSDH as the presenting manifestation of a spinal CSF leak is exceptionally rare and remains poorly characterized. The findings support inclusion of spinal CSF leaks in the differential diagnosis of unexplained or recurrent non-traumatic subdural hematomas and emphasize the importance of targeted spinal diagnostics and definitive leak closure.
